# Estrogen Biosynthesis and Action in Ovarian Cancer

**DOI:** 10.3389/fendo.2014.00192

**Published:** 2014-11-12

**Authors:** Felicitas Mungenast, Theresia Thalhammer

**Affiliations:** ^1^Department of Pathophysiology and Allergy Research, Center for Pathophysiology, Infectiology and Immunology, Medical University of Vienna, Vienna, Austria

**Keywords:** ovarian cancer, estrogen synthesis, estrogen sulfotransferase, estrogen sulfatase, estrogen receptor alpha/beta, G-protein-coupled estrogen receptor, progesterone

## Abstract

Ovarian cancer is still the deadliest of all gynecologic malignancies in women worldwide. This is attributed to two main features of these tumors, namely, (i) a diagnosis at an advanced tumor stage, and, (ii) the rapid onset of resistance to standard chemotherapy after an initial successful therapy with platin- and taxol-derivatives. Therefore, novel targets for an early diagnosis and better treatment options for these tumors are urgently needed. Epidemiological data show that induction and biology of ovarian cancer is related to life-time estrogen exposure. Also experimental data reveal that ovarian cancer cells share a number of estrogen regulated pathways with other hormone-dependent cancers, e.g., breast and endometrial cancer. However, ovarian cancer is a heterogeneous disease and the subtypes are quite different with respect to mutations, origins, behaviors, markers, and prognosis and respond differently to standard chemotherapy. Therefore, a characterization of ovarian cancer subtypes may lead to better treatment options for the various subtypes and in particular for the most frequently observed high-grade serous ovarian carcinoma. For this intention, further studies on estrogen-related pathways and estrogen formation in ovarian cancer cells are warranted. The review gives an overview on ovarian cancer subtypes and explains the role of estrogen in ovarian cancer. Furthermore, enzymes active to synthesize and metabolize estrogens are described and strategies to target these pathways are discussed.

## General Introduction

Currently, ovarian cancer is the fifth most common cancer in women in industrialized countries. It is mainly a disease of postmenopausal women, because more than 80% of all cases are being diagnosed in women older than 50 years. Although a falling rate of new ovarian cancer cases of 1.1% each year and an increase in the relative 5-year survival time from 33.6% in 1975 to 45.2% in 2010 was observed in industrialized countries, ovarian cancer is still the deadliest of all gynecologic malignancies worldwide. The alarming data are greatly attributed to the generally late diagnosis of the disease. More than 80% of all newly detected cases are advanced epithelial ovarian cancers (EOC) with peritoneal metastases and/or metastases in distant organs [Féderation Internationale de Gynécologie et d’Obstétrique (FIGO) stage III–IV]. The FIGO staging system was established by the FIGO and is most commonly used in EOC diagnosis (1). Effective preventative measures and reliable screening tools for an early detection are not yet available. Although the majority of women experience a variety of non-specific symptoms in the year before diagnosis, the disease is not commonly recognized until the tumor reaches an advanced stage. Another problem is the early development of resistance to the standard chemotherapy regimens with cisplatin/oxaliplatin in combination with paclitaxel. This leads to an early relapse and tumor progression. Considering these problems, it is clear that reliable diagnostic tools for an early detection of these cancers and more treatment options are urgently needed (2–4).

Epidemiological data show that induction and biology of ovarian cancer is related to estrogen exposure and metabolism. Experimental data demonstrated that ovarian cancer cells share a number of estrogen regulated pathways with other hormone-dependent cancers such as breast and endometrial cancer. Such pathways were studied in more details already in these tumors (5–7).

This review gives an overview on ovarian cancer heterogeneity and estrogen-related mechanisms in ovarian cancer biology. Thereby, data on enzymes active to synthesize and inactivate estrogens as well as on estrogen receptors (ER) are shown and strategies to target these pathways are discussed.

## Ovarian Cancer as a Heterogeneous Disease

### Classification

More and more, the classification of the heterogeneous group of ovarian carcinomas is coming into focus of research and ovarian cancer subtypes with different behaviors, mutations, origins, markers, and prognosis are characterized at a molecular level for their molecular signature. Since subtypes of ovarian cancer respond differently to the common therapies, a characterization of the tumor type is very important for a successful treatment (8).

Epithelial ovarian cancer is the most frequent tumor in the ovary as up to 98% of all cases are classified as EOCs. To this group belongs the most common and also the most deadly ovarian cancer, the high-grade serous ovarian cancer (HGSC), showing a frequency of 70%. It is followed by endometrial ovarian carcinomas (EC) and clear cell carcinomas (CCC) with frequencies of 10% for each entity, low-grade serous ovarian carcinomas (LGSC) with 5%, and mucinous carcinomas (MC) with 3% frequency (9). Other tumors, e.g., stromal cord tumors, teratomas, etc., are only rarely observed (10).

A summary of the predicted origins of different ovarian cancer subtypes and the mutations frequently found in these tumor types is given in Table 1.

**Table 1:** **Origins and significant mutations of the EOC subtypes**.

EOC subtypes	Predicted origin	Frequent Mutations
HGSC (high-grade serous carcinoma)	STICs (serous tubal intraepithelial carcinoma)	*BRCA1/2, p53, NF1, CDK12*, chromosomal instability
LGSC (low-grade serous carcinoma)	Borderline tumors of the ovary	*BRAF, KRAS*
EC (endometrial carcinoma)	Atypical endometriosis, uterus	*CTNNB1, PTEN, ARID1A*
MC (mucinous carcinoma)		*KRAS*, *HER2*
CCC (clear cell carcinoma)	Atypical endometriosis, uterus	*ARID1A*

**BRCA1/2*, breast cancer 1/2, early onset; *NF1*, neurofibromin 1; *BRAF*, BRaf proto-oncogene, serine/threonine kinase; *KRAS*, Kirsten rat sarcoma viral oncogene homolog; *CTNNB1*, catenin (cadherin-associated protein), beta 1; *PTEN*, phosphatase and tensin homolog; *ARID1A*, AT rich interactive domain 1A, *HER2*, tyrosine kinase-type cell surface receptor HER2*.

*Singer et al. (11), Shih Ie and Kurman (12), Köbel et al. (13), and Gilks and Prat (9)*.

Several immunohistochemical and genetic analyses have been done to detect differences and identify features of ovarian cancer subtypes. Köbel et al. (13) did biomarker analyses and came to the conclusion that some biomarkers, e.g., Ki-67 as a cell proliferation marker, Williams tumor protein 1 and also CA125 show significant differences in expression rates between the subtypes. Therefore, they can be used as subtype specific biomarkers.

The serous subtypes are classified as low-grade (LGSC) and high-grade (HGSC). LGSC and HGSC represent two distinct tumor types with a different underlying pathogenesis rather than low-grade and high-grade variants of the same neoplasm. Both are usually at advanced stage at the time of diagnosis (FIGO stage III or IV). *BRAF* (*v-raf murine sarcoma viral oncogene homolog B*) and *KRAS* (*Kirsten rat sarcoma viral oncogene homolog*) mutations are important molecular events in LGSC, while HGSC as the most common subtype of ovarian cancer is almost always associated with *p53* mutations. HGSC arises from the epithelium of the distal fallopian tube and not from the ovarian epithelial cells (9). In contrast to the serous tumors, EC and CCC are typically present as low-stage neoplasms and usually arise from endometriosis. Primary ovarian MCs are almost always unilateral and FIGO stage I tumors. This group consists mainly of so-called intestinal or enteric type MCs. Generally, MCs arise in a step-wise manner from a pre-existing mucinous cystadenoma or mucinous borderline tumor.

Another system classifies different ovarian cancer subtypes (mentioned above) into type I and type II tumors. Type I tumors (LGSC, low-grade EC, CCC, and MC) are generally slow growing and genetically more stable than type II tumors. A step-wise progression from a benign precursor lesion (endometriosis in the case of endometrioid tumors) to borderline tumors and next to the invasive tumors is characterized by genetic aberrations targeting specific cell signaling pathways, e.g., *KRAS* or *BRAF* mutations. Type II tumors (HGSC, high-grade EC, and undifferentiated carcinomas) are clinically aggressive and exhibit high genetic instability with frequent *p53* mutations (14).

### Serous ovarian carcinoma subtypes

#### High-grade serous carcinoma

High-grade serous ovarian carcinoma is a highly aggressive tumor, which is usually detected in an advanced stage. After initially responding to standard platin- and taxane-based chemotherapy, the majority of patients will experience recurrence and develop resistance to therapeutic drugs within 24 months (8, 15). Early peritoneal metastasis is also common (16). The pathological morphology of HGSC is heterogeneous showing a papillary, glandular, or solid architecture. The tumor cells form large multi-layered epithelial areas, which are surrounded by tumor stroma. Mononuclear giant cells with large nuclei are commonly found in these tumors (13).

In contrast to LGSCs, HGSCs have a very high mitotic rate and usually carry mutations in the *p53* gene. HGSCs are characterized by a high chromosomal instability. Loss of function mutations in the *breast cancer type susceptibility genes 1 and 2* (*BRCA1/2*) are also associated with HGSC. Somatic mutations in, e.g., *neurofibromin (NF1)* and *cyclin-dependent kinase (CDK)12* genes are common (9, 12, 16).

While previous models predict that HGSC develops from inclusion cysts of the ovarian epithelium, it is now agreed that HGSC arises from the serous tubal intraepithelial carcinomas (STICs). The latter develop from cells on the junction of the fallopian tube epithelium with the mesothelium of the tubal serosa. Cells there undergo malignant transformation and metastasize to the nearby ovary and later into the surrounding pelvic peritoneum.

Serous tubal intraepithelial carcinomas have mostly the same *p53* mutations, express the same oncogenes and also have similar phenotypic characteristics as HGSCs (15). STICs are found in 67% of all HGSC cases (17). They are also associated with *BRCA1/2* mutations (18).

High-grade serous ovarian cancer tumors in patients with mutated *BRCA1/2* have a more aggressive behavior and high-grade histology, but they are frequently responsive to chemotherapy. In many cases, their high sensitivity to the platinum-based regimens, may lead to a slightly improved 5-year survival (19).

#### Low-grade serous ovarian carcinoma

Low-grade serous ovarian cancer is rather rare with <5% of all EOCs. LGSCs are thought to develop stepwise from benign serous cystadenomas via the formation of serous borderline tumors to the final carcinoma. However, LGSCs rarely transform to HGSC tumors (9, 20). If LGSCs are detected at an earlier stage, the prognosis after treatment is favorable. Even for patients with advanced stage tumors, the 5-year survival is longer than that for HGSC patients, although LGSCs are quite resistant to conventional chemotherapy. Similar to HGSC, this subtype often spreads intraperitoneally (21). In the histological picture, micropapillary structures and psammoma bodies (which are calcium incorporations that are formed from necrotic tumor cells) are frequently seen. LGSC cells have rather uniform nuclei and a much lower mitotic rate than HGSC tumor cells. Genetically, there is less chromosomal instability in LGSC than in HGSC. However, the presence of *BRAF* and *KRAS* mutations, as well as mutations in other genes (Table 1) is common (11, 16).

## Ovarian Cancer as a Hormone-Dependent Cancer

### Epidemiological data

There is strong epidemiological evidence that etiology, pathogenesis, and progression of ovarian cancers are greatly dependent on the activity of estrogens. Furthermore, the balance between estrogen and progesterone is critical for the formation of ovarian cancers (22).

Statistical analyses show that the incidence of ovarian cancer is much higher in industrial countries than in developing countries. The birth rates in industrial countries are low compared to developing countries (23). There is strong evidence that reproductive factors including multiple pregnancies, breastfeeding, and use of oral conceptive pill (OCP) protect against ovarian cancer. With each pregnancy, the risk of developing ovarian cancer decreases by 10–16% and a pregnancy at the age of 35 years is twice as protective as at the age of 25 years (24, 25). Also, a significant protective effect is seen in women that do breastfeeding for more than 18 months (26, 27). Similarly, application of OCP for more than 3 years causes a 30–50% reduced risk of developing ovarian cancer (28).

In contrast to these protective factors, women with an early first period and a late menopause as well as women that receive drugs for the treatment of infertility (gonadotropin releasing-hormone antagonists or clomiphene) have an increased risk of developing ovarian cancers. The latter is thought to be caused by high concentrations of estrogen after stimulation of the sex-steroid hormone synthesis in the ovary (29). Also, application of hormone replacement therapy (HRT) was found to be a risk factor for ovarian cancer. An approximately 22% increased risk of ovarian cancer over 5 years was seen in postmenopausal women using unopposed estrogen as HRT. The risk was still significantly increased (by approximately 10%) by the application of a combination of estrogen and progestin (30, 31). Data from a study in a large patient cohort in England revealed that the incidence of ovarian cancer increased with longer duration of HRT therapy, especially if HRT was taken for 10 or more years. There was a higher relative risk for developing EOCs rather than MCs, ECs, or CCCs. But the composition of the HRT did not influence the risk (32). A more recent study revealed that women taking unopposed oral estrogen therapy have an increased risk of HGSCs, LGSCs, and ECs. Only the risk for MCs was decreased. Similar, an increased risk for serous carcinomas and ECs was found in women receiving an estrogen/progestin combination as HRT (33). On the other hand, women with previous HRT have a better prognosis when diagnosed with ovarian cancer. They are more likely to be diagnosed at younger age and lower tumor stage allowing a complete surgical removal of the tumor mass (complete debulking without any signs of a residual tumor mass). An increased overall survival, specifically in the subgroup of patients, which had a complete debulking, was found in these studies. No correlation was seen with the progression free survival (34). Data from another study, however, showed that if HRT was administered following tumor debulking, the prognosis remained unchanged. Furthermore, the survival time was independent on the expression of estrogen and progesterone receptors (PGR) in the cancer tissues. This study was done with a small number of patients only, and therefore, data may not be representative for a larger cohort of ovarian cancer patients (35). After tumor operation, especially younger ovarian cancer patients may suffer from estrogen withdrawal symptoms, and therefore, they will consider HRT treatment. Since data are not clear yet, studies with more patients suffering from different types of ovarian cancer are urgently needed.

### Ovarian cancer hypothesis

To explain the effects of estrogens in the etiology of ovarian cancer, different hypothesis are available. The “incessant ovulation hypothesis” was developed already in 1971, but the more recent “incessant menstruation hypothesis” is now favored (36–38).

#### The incessant ovulation hypothesis

This hypothesis attributes ovarian cancer formation to repetitive wounding during ovulation and the subsequent activation of repair mechanisms. These processes are associated with an increased number of mutations accumulating in epithelial cells. This finally drives tumor formation and progression (39).

The association between sex steroids and cancer can be explained by processes that take place during the menstrual cycles, in which the ovarian surface epithelium (OSE) plays pivotal roles during ovulation and postovulatory wound repair. Indeed, most of the total proliferative activity of the OSE is related to ovulation repair and formation of the *corpus luteum*. In the menstrual cycle, the OSE covering growing follicles enters into the proliferative phase during pro-estrus/estrus transition. After the ovulation, the proliferation rate of OSE cells covering the newly formed *corpus luteum* decreases. Also, the exposure of the OSE to high doses of the gonadotropins luteinizing hormone (LH) and follicle stimulating hormone (FSH) during the menstrual cycle promotes cell proliferation and tumor growth (40). As a positive effect, progesterone, which is increased during pregnancy and during OCP application, promotes clearing of transformed cells from the ovarian surface epithelial layers (37, 41).

#### The incessant menstruation hypothesis

High-grade serous ovarian cancer are sought to derive from cells in the fimbriae of the fallopian tubes, which are floating in bloody peritoneal fluid. Thereby, they are exposed to iron-induced oxidative stress derived from retrograde menstruation. The genotoxic effect of reactive oxygen species, generated from hemolysis of erythrocytes by pelvic macrophages would explain the distal site of tubal intraepithelial neoplasia (37, 38, 41).

### Estrogens and ovarian cancer

At the cellular level, tumor promoting effects of estrogen are conferred in a (i) receptor-dependent and (ii) -independent way.

**Figure 1:**
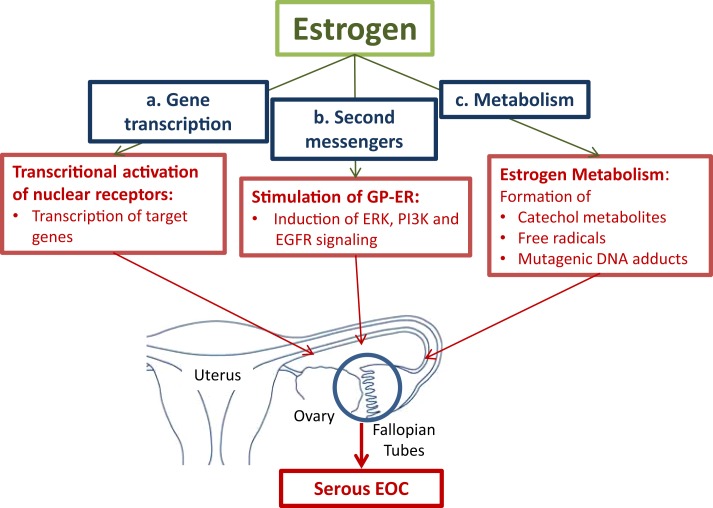
**Pathways for estrogen to convert tumor promoting effects in cells in the fallopian tubes and the ovaries**. **(a.)** Activation of the nuclear estrogen receptor-α (ER-α) leads to the transcriptional activation of estrogen-responsive genes, which stimulate cell proliferation. **(b.)** Binding to membrane-bound G-protein-coupled estrogen receptor (GPER) activates second messenger systems. In cancer cells, estrogen induces extracellular-signal regulated kinase (ERK), phosphoinositide 3-kinase (PI3K), and epidermal growth factor receptor (EGFR) leading to enhanced cell proliferation. **(c.)** The formation of reactive metabolites leads to the generation of mutagenic DNA adducts. Free radicals from the metabolic activation of estrogens will cause mutations. Accumulation of mutations will lead to neoplastic transformation of proliferating cells (10, 42, 44–46)

i) Receptor-dependent ways: binding of estrogen to the nuclear estrogen receptor-α (ER-α) leads to the transcriptional activation of estrogen-responsive genes, which provide signaling systems for cell division and differentiation. Among these genes are proto-oncogenes, such as c-fos, c-myc, and HER2/neu; cell cycle regulating cyclins, growth factors, and others (42). Binding to membrane-bound G-protein-coupled estrogen receptor (GPER, formerly known as GPR30) activates second messenger systems. Thereby, GPER confers rapid non-genomic effects of estrogens (43).
ii) In a receptor independent way, formation of reactive metabolites via cytochrome P450 enzymes (CYPs) may lead to the generation of mutagenic DNA adducts. Free radicals generated by the metabolic activation of estrogens cause mutations. Accumulation of mutations in various genes in cells in the fallopian tubes and in the ovary will lead to the neoplastic transformation of cells (Figure 1) (10, 44, 45).

#### Description of pathways

i) Transcriptional effects of estrogens on target genes are mediated by activation of nuclear receptors, the estrogen receptor-α (ER-α), and estrogen receptor-β (ER-β). Upon binding of estrogens to ER-α, transcription of a battery of genes, which stimulate cell proliferation, is induced. Enhanced proliferation is associated with an increased risk of mutations that accumulate in cells finally leading to malignant transformation. ER-β was found to counteract the growth stimulating effects of ER-α in ovarian cancer cells (47).
  Another member of the nuclear receptor superfamily, which confers estrogenic effects in ovarian cancer is the estrogen-related receptor-α (ERR-α). It is known to regulate metabolic homeostasis under conditions of high energy demand, e.g., in brown adipocytes. The increased expression and activity of ERR-α was associated with a less favorable clinical outcome of ovarian cancer (42).
  Estrogens also promote tumor progression by influencing signaling pathways. Via the seven-transmembrane spanning G-protein-coupled receptor, named GPER, estrogen rapidly activates the extracellular signal-regulated kinases (ERK)-1 and ERK-2, and confers estrogenic effects to cells devote of the classical nuclear ER (48). A recent study in endometrial cancer cells showed that GPER mediates the estrogen stimulated induction of the kinases ERK-1 and -2 and the phosphatidylinositol-4, 5-bisphosphate 3-kinase (PI3K) via activation of matrix metalloproteinase. This is followed by the subsequent transactivation of the epidermal growth factor receptor (EGFR) (46).
ii) Metabolism of estrogen may cause DNA damage by the formation of mutagenic purinergic DNA adducts and by generation of free radicals from the metabolic activation to reactive catechol estrogens. Catechol estrogens are formed by aromatic hydroxylation of primary estrogens at either the C-2 or C-4 position. The catechol metabolites are inactivated by their conjugation with active sulfate or uridine-diphosphate (UDP)-glucuronic acid by steroid sulfotransferases (SULTs) and UDP-glucuronosyltransferases, respectively. Before their conjugation to more water-soluble metabolites, hydroxylation of the steroid moiety via specific CYP isoenzymes, namely CYP1A1 and CYP1A2, which catalyze hydroxylation in position 2, and CYP1B1, which is an estrogen 4-hydroxylase, occurs (44). Especially, 4-hydroxyestrogens can be oxidized to quinone intermediates, which react with purine bases of the DNA. This results in depurinating adducts, which generate highly mutagenic, apurinic sites. 2-hydroxyestrogens produce less genotoxic DNA adducts. Studies in rodents demonstrated that E2 and E1, as well as their catechol metabolites, in particular 4-hydroxy E2/E1 (4-OH E2/E1), have carcinogenic effects. In a redox cycle, 4-OH E2/E1 is converted to the quinone derivatives. The conversion back to 4-OH E2/E1 is associated with the formation of oxygen radicals. DNA mutations caused by free radicals will lead to the neoplastic transformation of cells (45).

### Estrogen and BRCA1/2 mutations

Breast and ovarian cancer mostly arise sporadically, but a small number of cases (approximately 10%) of these cancers are associated with mutations in *BRCA1/2* genes. Defects in the DNA damage response or in the DNA repair pathways in patients with the *BRCA1/2* mutation are responsible for the high penetrance of these cancers in the breast and/or ovary. It was shown that carriers of *BRCA1/2* mutations have also increased levels of estrogen, which may trigger breast and ovarian cancers (49). Indeed, in premenopausal patients with *BRCA1/2* mutations, removal of both ovaries and of the fallopian tubes reduces the risk of these cancers (50).

## Estrogen Synthesis in Ovarian Cancer Cells

During the reproductive years, ovaries produce and release progesterone and the estrogens estrone (E1), 17β-estradiol (E2), and estriol (E3).

E2, the most active form of natural estrogens, together with progesterone is critical for normal uterine function, establishment and maintenance of pregnancy, and mammary gland development. Furthermore, it is responsible for endocrine, paracrine, and autocrine actions in various tissues and organs. Ovarian steroid hormone formation takes place in the ovarian granulosa and theca cells, which work in a collaborative way for the synthesis after stimulation by the gonadotropins LH and FSH. Theca cells respond to LH signaling by increasing the expression of steroid synthesizing enzymes for the transformation of cholesterol to the androgens (5-androstenedione and testosterone). Granulosa cells respond to FSH signaling by stimulating the expression of enzymes for the synthesis of estrogens (E2 and E1) from androgen precursors (51).

While in premenopausal women, the main part of active estrogens derives from the synthesis in the ovary, after the menopause, estrogens are formed locally in various tissues such as in liver, brain, and adipose tissue. There, E2 is produced from circulating androgen and estrogen precursors. These precursors are bound to sex-steroid binding globulins in the blood. They are transported to the ovary, where they are taken up into ovarian epithelial cells by transporters, e.g., from the family of organic anion transporting peptides (OATPs) (52). The importance of the visceral adipose tissue of postmenopausal women for E2 production is reflected by the high concentrations of estrone-sulfate (E1-S), 5-androstendione, and dehydroepiandrosterone-sulfate (DHEA-S) in these cells. The local concentrations are up to 60× higher than in serum, while E2 and testosterone levels are increased by sevenfold only (53, 54).

The biological activity of estrogen is regulated independent on the expression and activity of receptors by the expression and function of steroid (estrogen)-metabolizing enzymes expressed locally at the target tissues. Among these enzymes, aromatase (CYP19A1), steroid sulfatase (STS), and 17β-hydroxysteroid dehydrogenases (17β-HSD) are highly important.

**Figure 2:**
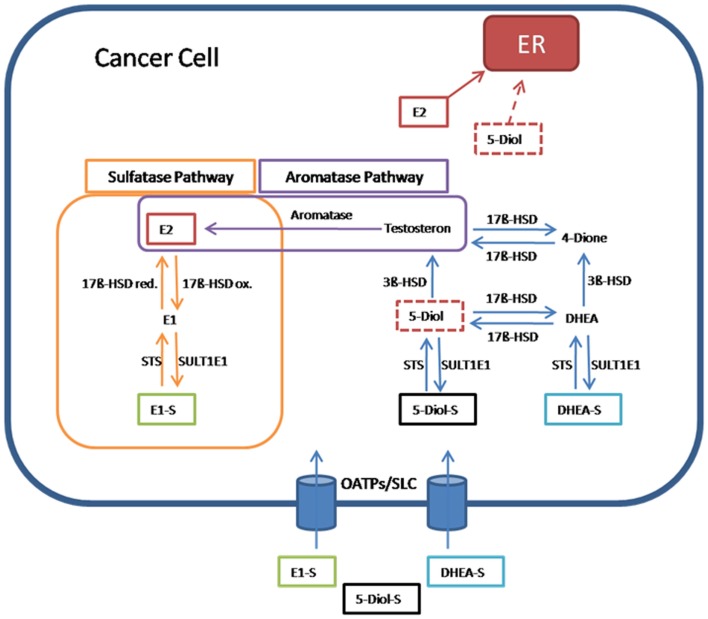
**Pathway for 17β-estradiol formation in cancer cells**. Estrone-sulfate (E1-S), a precursor for the most active estrogen 17β-estradiol (E2), androstenediol (5-Diol-S), and dehydroepiandrosterone sulfate (DHEA-S) are taken up from the blood into cancer cells by transporters from the organic anion transporting polypeptide family (OATPs) and other members of the solute carriers (SLCs). In the sulfatase pathway, E1-S is converted through steroid sulfatase (STS) to estrone (E1), which is transformed via the reductive 17β-hydroxysteroid dehydrogenases (17β-HSD red) to E2. E2 as the most active estrogen binds and activates estrogen receptors (ERs). In the reverse pathway from E2 to E1-S, the oxidative 17β-hydroxysteroid dehydrogenases (17β-HSDox) convert E2 to E1. The estrogen sulfotransferase SULT1E1 inactivates estrogens by adding sulfate to hydroxyl-groups on the steroid ring. In the aromatase pathway, E2 is produced from testosterone by the aromatase. Testosterone itself is formed from 5-androstenediol (5-Diol) via 3β-HSD. 5-Diol is generated by the removal of the sulfate from 5-Diol-S via STS. Also 5-Diol can activate ER, but with lower affinity than E2. In an alternative way, testosterone is derived from the conversion of DHEA-S to androstenedione (4-Dione) via DHEA. Finally, 17β-HSD transforms 4-Dione to testosterone (55).

In hormone-dependent cancer, formation of the biological most active estrogen E2 from steroid precursors is mediated via the aromatase and the sulfatase pathway (Figure 2).

In the aromatase pathway, steroid precursors derived from the circulation, like dehydroepiandrosterone (DHEA), are first converted to testosterone and, subsequently, by the aromatase, E2 is generated.

In the sulfatase pathway, the sulfate moiety is removed by STS from the inactive E1-S to form the active estrogen E1. E1 is converted to E2 by the reducing 17β-HSD isoenzymes. In the inactivating pathway, E2 is oxidized to E1 by 17β-HSD enzymes. E1, which exerts also estrogenic effects via binding to ERs, can be inactivated by estrogen sulfotransferase (SULT1E1). The resulting inactive E1-S can again become activated by the removal of sulfate (7, 55).

The precursor of all steroid hormones is cholesterol, which is mainly derived from the synthesis, particularly in the liver, or from the nutrition. Cholesterol is converted over a few steps to pregnenolone, and next to progesterone. Progesterone acts as the starting point for all steroid hormones in the adrenal cortex and in various other peripheral organs, e.g., the liver. The steroid hormone precursors DHEA, its sulfate metabolite DHEA-S, 5α-androstenediol sulfate (5-Diol-S), and E1-S are also synthesized in large quantity there. They are released into the circulation.

In the group of postmenopausal women, the levels of E2 and progesterone are up to 90% lower than those from premenopausal women. However, the concentration of other steroid hormones (DHEA, 5-Diol-S) is similar in pre- and postmenopausal women. After the menopause, E1-S is the most abundant estrogen in the peripheral blood of women. Ten to thirty percent of estrogens in serum are bound to sex-steroid binding globulins in order to provide a reservoir for peripheral formation of E2. These proteins are synthesized in the liver after the stimulation with estrogen and thyroid hormones. Progesterone inhibits their production (56, 57).

### Estrogen synthesis in hormone-dependent cancers

Epidemiological and experimental studies showed that higher endogenous estrogen exposure through early menarche (<12 years), late menopause (>55 years), nulliparity, obesity (postmenopausal), use of postmenopausal HRT, and increased plasma E2 levels lead to an increased risk of breast cancer. Of particular importance is the circulating inactive plasma estrogen precursor E1-S, which has been reported to serve as the predominant source for tumor E2 in postmenopausal patients with breast cancer. E1-S is derived from peripheral tissues, including the adrenal gland, adipocytes, liver, muscle, skin, and bone (54). Following its cellular uptake by transporters of the OATPs family (52), E1-S is desulfonated to E1 by STS and E1 is further converted to E2 by 17β-HSDs. This intracellular production of E2 stimulates the proliferation of estrogen-dependent tumor cells (58). As compared to the other sources of unconjugated estrogens (which act as precursors for the aromatase pathway), E1-S (precursor for the sulfatase pathway) has about 5–10 times higher plasma circulating levels than other estrogens (59). Moreover, sulfatase activity is 130–200 times higher than aromatase activity (60) and the concentration of sulfatase is three times higher in breast cancer tissues than normal tissues (61). Two-thirds of all breast cancers have a positive ER status and as consequence, they are sensitive to estrogens. These tumors respond well to hormone therapy (62). Also in endometrial cancer, biosynthesis of active estrogen is achieved from E1-S, which is transported into endometrial cells, where it is converted to E2 by STS and 17β-HSDs (63).

## Enzymes in the Estrogen Metabolism Pathway

### Steroid sulfatase

Steroid sulfatase is a member of the arylsulfatase family, which is known for the hydrolysis of sulfate ester bonds in a wide range of substrates. The corresponding gene is located on the X-chromosome and contains 10 exons. The group of Hernandez-Guzman et al. (64) was the first to isolate STS from the human placenta, to purify and crystallize it. This enzyme consists of two membrane-spanning hydrophobic alpha helices that are arranged anti-parallel. The amino acid proline on position 212 acts on the cytosolic side of the endoplasmatic reticulum membrane as turning point. STS is present in the ER of many tissues and is especially high in ovarian granulose cells (65, 66). It plays an essential role in the synthesis of E2, by converting E1-S into active E1 (Figure 2).

### Estrogen sulfotransferase

Estrogen sulfotransferase belongs to the family of SULTs, phase II detoxification enzymes. Ten isoforms of SULT are known to be expressed in humans. These enzymes have a wide range of substrates including hydroxysteroids, thyroid hormones, phenols, arylamines, and primary alcohols. Because SULT1E1 has the highest affinity of all SULTs for E1 and E2, it is also called estrogen specific sulfotransferase. It catalyzes the transfer of a sulfuryl group from 3′-phosphoadenosine 5′-phosphosulfate (PAPS) to nucleophilic groups of SULT1E1 substrates. PAPS are synthesized by PAPS synthesizing enzymes (PAPS synthetases).

Estrogen sulfotransferase acts in an antagonistic way to STS by converting E1 to E1-S. Therefore, it is inactivating the estrogen. The corresponding gene is located on chromosome 4q3.12, has eight exons and a length of 20 kB. SULT1E1 is present in various tissues, including liver, testis, mammary epithelium cells, and endometrium (67–69).

### 17β-hydroxysteroid dehydrogenases

17β-HSDs form a family of enzymes (14 isoforms in vertebrates) that catalyze the conversion between highly active 17β-hydroxy steroid hormones and 17-keto steroid hormones with lower or even missing activity. 17β-HSD isoenzymes have different enzymatic properties and a characteristic cell-specific expression pattern according to their different physiological functions. They are multifunctional and modulate other steroid structures as well. Among their substrates are bile acids, retinoids, fatty acids, and others.

The reductive 17β2-HSD isoenzyme activates E1 to E2 and 4-Dione to testosterone. Also the isoforms 7 and 12 are reductive estrogenic enzymes. In contrast, isoforms 4, 8, 10, and 14 are oxidative enzymes responsible for E2 inactivation (63).

In the normal ovary, 17β-HSDs are detected in granulosa cells of developing follicles, but not in the normal OSE. However, a variety of EOC have been reported to be positive for different 17β-HSD isoenzymes.

For example, a recent study showed that reducing 17β12-HSD is overexpressed in many human carcinomas including ovarian carcinoma, while it is not present in the normal OSE. Importantly, patients with EOC tumors with a weak or moderate expression of 17β12-HSD had a better overall survival than those with strongly 17β12-HSD positive tumors (55).

## Estrogen Receptors

### Estrogen receptors-α/-β

Currently, two isoforms of the nuclear ER, namely ER-α and ER-β are known. In 1996, Kuiper et al. (70) cloned the second ER, named ER-β from rat testis. As a consequence, ER was renamed to ER-α. The two ERs are encoded by the *ESR1* and *ESR2* genes. Alternative promoter usage and splicing produces various transcript variants. However, expression pattern and function of many of these variants has not been determined so far.

The ERs are members of the nuclear receptor superfamily of steroid receptors and function as ligand inducible transcription factors (71). The nuclear receptor superfamily structure is defined through five different domains, namely A/B, C, D, E, and F. Each domain fulfills another obligation, which is essential for the correct function of the receptor. The A/B domain, also named N-terminal domain includes a ligand independent activation function (AF-1). This domain also has a rather poor (20%) homology with the other ER isoform (72). This gives a hint that this region takes mainly part to the ER subtype specific actions on target genes. Moreover ER-α seems to have a stronger corresponding AF-1 function than ER-β (73). The DNA-binding domain C is responsible for specific DNA-binding and receptor dimerization. This highly conserved domain shows a high homology between ER-α and ER-β. The D-domain is a flexible hinge with the ligand-binding domain and is called “Hinge domain.” ER-α shares only 30% homology in this domain with ER-β. It contains a nuclear localization signal, which is essential for the transport of ERs to the nucleus. The ligand-binding E-domain is responsible for hormone-dependent activation (AF-2), ligand binding, and together with the DNA-binding domain for receptor dimerization. ER-α and ER-β show a homology of 55% in the ligand-binding domain (74, 75). The amino acid sequence of the ligand-binding cavity varies only in two positions, but this leads to a significant smaller cavity in ER-β. This may be an advantage for receptor subtype specific drugs (76, 77). The function of the F domain is still not clear. A dimerization of ER is necessary for transcriptional activation. Either a homodimer of one ER isoform or a heterodimer of ER-α and ER-β is active (73). The regulation of the transcription of target genes is achieved by binding of the receptors to estrogen response elements in target genes. This is followed by recruitment of a variety of coregulators to alter chromatin structure and facilitate the recruitment of the RNA polymerase II transcriptional machinery (78).

Estrogen receptors mediate many estrogenic effects on gene regulation and, therefore, ERs are essential for various developmental and functional processes in several tissues/cells (79). ERs are important for sexual development and reproductive function, but also play a role in other tissues such as bone.

ER-α is the predominant ER in the uterus, mammary gland, testis, pituitary, liver, kidney, heart, and skeletal muscle (80–82). Some ER-α target genes mediate proliferation and cell survival (83). Importantly, sustained estrogenic exposure and activation of ER-α may increase the risk and/or the progression of various cancers, including cancers in the breast and endometrium.

The ER-β gene consists of eight exons and through alternative splicing five isoform, namely ER-β1-5 are generated by deletion of one or more exons. Human ER-β1 protein has a length of 530 amino acids (84, 85). ER-β is predominantly expressed in ovary and prostate (80–82). In contrast to ER-α, ER-β activates antiproliferative and pro-apoptotic pathways in many cancer cells (83).

### The membrane-bound G-protein-coupled estrogen receptor

G-protein-coupled estrogen receptor functions as a G coupled plasma membrane-associated receptor and works independent of nuclear ERs. It binds various estrogens including E2, E1 and E3, ER antagonists, phytoestrogens, and xenoestrogens (86). GPER, which mediates rapid estrogen signaling via stimulation of adenylyl cyclase, is expressed in normal ovary, where it regulates physiological processes such as follicle maturation. The receptor is expressed in many EOC samples. However, the prognostic impact of GPER expression in EOC is controversial. In an earlier study, GPER was seen to be preferentially expressed in “high risk” tumors with a worse prognosis (87). Later, no relation between GPER expression and survival rates of EOC patients was found (88). Recently, GPER expression was shown to correlate with gonadotropin (LH and FSH) receptors. Only in EOCs, which are negative for gonadotropin receptors, a higher GPER expression was associated with a more favorable outcome for the patients. These findings suggest that GPER may reduce ovarian cancer cell proliferation in the absence of gonadotropin signaling only (89). Therefore, synthetic agonists and antagonists for GPER, which are now available, might be tested in gonadotropin receptor-negative tumors of EOC patients (90).

## Progesterone and Progesterone Receptors

Progesterone is synthesized from cholesterol in the *corpus luteum*, follicles, placenta, and in other organs, e.g., the adrenal gland. During transport through the blood plasma, it is bound to cortisol binding globulin, because of its lipophilic nature and also to avoid degradation.

In the ovary, progesterone plays an important role in the follicle maturation and moreover, it is responsible for preparing the female genital tract for pregnancy. It also maintains the pregnancy after fertilization. Progesterone promotes the growth of the uterus musculature and changes the endometrium from a proliferative to a secretory tissue. It also decreases the myometrium activity during pregnancy and changes the quality and quantity of the cervix mucus, thus preventing the entrance of sperms into the uteri and the tubes. In the ovary, it works in concert with estrogen to promote follicle maturation, ovulation, and formation of the *corpus luteum*.

The effects of progesterone are mediated by the two members of nuclear PGR isoforms. Like other receptors of this family, PGR consists of four domains, namely the ligand-binding domain, the Hinge-Region as the flexible link between the ligand-binding domain and the DNA-binding domain, and the N-terminal domain (91). The two isoforms PR-A and PR-B are encoded by one gene but the transcription is carried out by two different promoters. Apart from the lack of a 164 amino-sequence at the N-terminal end in PR-A, the two PGRs are identical. PR-B is responsible for the transcriptional activation of progesterone responsive genes, but it can be inhibited by PR-A (92–95). PR-A and PR-B act either as homo- or heterodimers and both are synthesized in equal amounts in normal epithelial cells (96).

Progesterone as an antagonist of estrogen has an antiproliferative effect on specific cells. It acts in part by decreasing the production of ERs, and through activation of 17β-HSD and SULT1E1. Indeed, elevated PGR levels were associated with a significantly better survival rate in EOC patients as data from a recent meta-analysis showed (97).

## Estrogen Synthesizing Enzymes and Receptors in Ovarian Cancer

There is evidence that estrogens play a role in the progression of ovarian cancer. An overexpression of STS will lead to an increased level of E2 and this may contribute to cancer progression. In postmenopausal women, the local conversion from circulating steroid hormone precursors, e.g., E1-S and DHEA-S to active E2 could promote ovarian cancer progression.

Indeed, the expression levels of key enzymes vary between normal tissues and different subtypes of EOC. For example, STS was detected in 30% of serous carcinomas, in 70% of CCCs, and in 8% of MCs (98). Ovarian cancer studies further showed that a longer progression free survival is significantly associated with lower STS levels. That can be explained by the fact that through a high expression of STS, more E2 is synthesized (99). Similar effects of STS were also reported from breast cancer studies, where STS activity correlated with the E2 serum levels. On the opposite, high estrogen inactivating SULT1E1 levels were associated with smaller tumors, a better prognosis, and a negative lymph node status in ovarian cancer patients (100–103). In the same way, in breast cancer a high expression of SULT1E1 together with a decreased level of STS correlates with a better prognosis, smaller tumor size, and a negative lymph status at the time of diagnosis. Another evidence that SULT1E1 acts as a tumor suppressor is based on a study with xenograft models carried out by Xu et al. (69) showing that an overexpression of SULT1E1 inhibited estrogen-dependent cell growth and induced tumor cell apoptosis.

High E2 levels were often observed in ovarian cancer patients (102). E2 was shown to increase the mobility of ovarian cancer cells via the inhibition of cell–cell adhesion. This promotes metastasis (104), and a similar effect of E2 was also observed in breast cancer (105).

Mostly earlier studies in ovarian cancer patients reported that high levels of ER-α and low levels ER-β are associated with a worse prognosis (106–110). However, in other studies, high expression of ER-α was found to be associated with a better prognosis (111, 112). Also higher ER-β levels were significantly associated with longer disease-free survival and a longer overall survival in one study. The reduction of ER-β significantly correlated with the hyper-methylation of the ER-β promoter, causing an inhibition of gene translation (113). A proof for the protective effect of ER-β is that this isoform is the dominant ER isoform in healthy ovaries. But in all serous tumors and also in metastasis, ER-α is usually dominant, and ER-β expression is rather weak. This leads to the conclusion that the gradual reduction of ER-β during tumor progression (from normal to borderline to malign tissue to metastasis) is a continuous process and important for malignant transformation and cancer cell proliferation (114, 115).

Progesterone has been proved to decrease the proliferative effect of estrogens and inhibit inflammation and cancer penetration by suppressing ovulation. It also initiates apoptosis in tumor cells. PGR is a biomarker for a better prognosis and longer overall survival in ovarian cancer (116). This is in line with findings that PGR levels are significantly lower in benign, borderline, and malignant ovarian tumors than in healthy tissue (108, 117). Recently, a multi-center investigation in 2933 women with invasive EOCs showed that PGR and ER are positive prognostic biomarkers for ECs and HGSCs (118).

### Ovarian cancer and hormonal treatment

There is evidence that estrogen has an influence on the progression of the EOCs at least in some subgroups of patients (119). To combat estrogen stimulated tumor cell growth, selective estrogen receptor modulators (SERMs) that function as agonists or antagonists for ERs, have been developed. However, modulation of the tissue-specific expression of ER subtypes, expression of co-regulatory proteins, and varying ER conformational changes induced by ligand binding may change the activity of the hormonal therapy. The best studied SERM is tamoxifen that is highly effective to block the ER-signaling pathways. It prevents breast cancer recurrence in many patients with positive ER status. In postmenopausal patients failing tamoxifen therapy, the synthesis of estrogen can be blocked by aromatase inhibitors anastrozole, letrozole, and exemestane (120).

In ovarian cancer, the therapeutic value for SERMs and aromatase inhibitors to block tumor progression and recurrence is not thoroughly established, yet. Only small-scale studies were done so far. Additionally, in the few studies, the patients were not selected based on their hormone-receptor status (ER positive or negative) or age (pre- vs. postmenopausal). Moreover, in some studies, patients were only selected after resistance to standard chemotherapy. Later, the group of Tropé et al. (121) compared data from different studies with tamoxifen (in total a collective of 647 patients) and found a response rate ranging from 0 to 56%, with a mean response rate of 11%. The treatment with aromatase inhibitor letrozole showed a response rate ranging from 0 to 35.7%, with stable disease rates ranging from 20 to 42% (122–124). For anastrozole, response rates of 1.9–4.3% and stable disease rates of 42–61% were reported (125, 126).

Whether ovarian cancer patients, who express ERs and estrogen synthesizing enzymes such as STS and 17β-HSDs in their tumors, may have a better response rate to hormonal therapy, should be investigated. Nevertheless, inhibition of estrogen activating STS would offer a novel approach to combat ovarian cancer. Among already available STS inhibitors, the cyclopentane carboxylate derivate STX64 (irosustat) is currently undergoing clinical trials for therapy of prostate, endometrial and breast cancer. With this drug, serum levels of E1, E2, 4-Dione and DHEA were decreased and stable disease for up to 7 months was even seen in breast cancer patients with advanced disease. However, for a more efficient depletion of tumor estrogen, application of STS inhibitors together with an aromatase inhibitor could also be of benefit in postmenopausal women in order to block both, E2 formation via the aromatase and sulfatase pathway (57). Also, subgroups of ovarian cancers should be studied in different therapeutic settings. This would help to identify patients, for which hormonal therapy might offer an additional possibility to prevent recurrence of ovarian cancer.

## Summary

Ovarian cancer is the deadliest of all gynecological malignancies in women and these tumors are usually seen in women after the age of 50 years. The still poor prognosis for ovarian cancer is partly attributed to the fact that the diagnosis is usually made at a late stage, when the cancer has already spread to other organs. There are only limited options for a successful chemotherapeutic treatment so far and novel strategies are needed.

Several epidemiological and experimental data revealed that ovarian cancer shares many estrogen regulated pathways with other hormone-dependent cancers, e.g., breast cancer. Therefore, local estrogen synthesis from circulating steroid hormone precursors by steroid-forming and steroid-inactivating enzymes may be important to drive ovarian cancer progression in women after the menopause. Indeed, these enzymes and receptors were identified in ovarian cancer cells and their expression was shown to be related to clinical parameters. So far, such studies were mostly done in a small group of patients, which were not selected based on their age, ovarian cancer subtype, hormone-receptor status, and resistance to standard chemotherapy. Because ovarian cancer is a heterogeneous disease and tumors vary with respect to their origin, behavior, and prognosis, they may also differ in their sensitivity to hormonal therapy. At least in subgroups of patients, who express enzymes for estrogen biosynthesis and receptors for estrogen signaling in their tumors, hormonal therapy might offer an additional possibility to prevent recurrence of ovarian cancer.

The review explains the role of estrogen in ovarian cancer and it gives an overview on ovarian cancer subtypes. Furthermore, enzymes active to synthesize and metabolize estrogens as well as estrogen signaling pathways are described. Strategies to target these pathways are discussed.

## Conflict of Interest Statement

The authors declare that the research was conducted in the absence of any commercial or financial relationships that could be construed as a potential conflict of interest.

## Abbreviations

CCC, clear cell carcinoma; CYP, cytochrome P450 enzyme; DHEA(-S), dehydroepiandrosterone(-sulfate); EC, endometrial ovarian carcinoma; EOC, epithelial ovarian cancer; ER-α, estrogen receptor-α; ER-β, estrogen receptor-β; ERR-α, estrogen-related receptor-α; E1, estrone; E1-S, estrone-sulfate; E2, 17ß-estradiol; E3, estriol; FIGO, Féderation Internationale de Gynécologie et d’Obstétrique; FSH, follicle stimulating hormone; GPER, G-protein-coupled estrogen receptor; HGSC, high-grade serous ovarian cancer; HRT, hormone replacement therapy; LGSC, low-grade serous ovarian cancer; LH, luteinizing hormone; MC, mucinous carcinomas; OATP, organic anion transporting polypeptide; OCP, oral conceptive pill; OSE, ovarian surface epithelium; PGR, progesterone receptor; SERM, selective estrogen receptor modulators; STIC, serous tubal intraepithelial carcinoma; STS, steroid sulfatase; SULT, sulfotransferase; SULT1E1, estrogen sulfotransferase; 4-OH-E1/E2, 4-hydroxy E2/E1; 5-Diol-S, 5α-androstenediol sulfate; 17β-HSD, 17β-hydroxysteroid dehydrogenases.
